# Diagnostic Value of CEUS LI-RADS Version 2017 in Differentiating AFP-Negative Hepatocellular Carcinoma from Other Primary Malignancies of the Liver

**DOI:** 10.3390/diagnostics11122250

**Published:** 2021-12-01

**Authors:** Peihua Wang, Fang Nie, Tiantian Dong, Dan Yang, Ting Liu, Guojuan Wang

**Affiliations:** Medical Center of Ultrasound, Lanzhou University Second Hospital, Lanzhou 730030, China; wangph19@lzu.edu.cn (P.W.); dongtt20@lzu.edu.cn (T.D.); yangd17@lzu.edu.cn (D.Y.); liut15@lzu.edu.cn (T.L.); wanggj19@lzu.edu.cn (G.W.)

**Keywords:** contrast-enhanced ultrasound, liver imaging reporting and data system, liver tumor, alpha-fetoprotein

## Abstract

Purpose: To explore the diagnostic value of Contrast-enhanced Ultrasound Liver Imaging Reporting and Data System version 2017 (CEUS LI-RADS v2017) in differentiating alpha-fetoprotein (AFP)-negative hepatocellular carcinoma (HCC) from other primary malignancies (OM) of the liver. Methods: The data of 99 patients with primary liver malignant tumors confirmed by surgical pathology and AFP-negative from January 2018 to January 2021 were retrospectively analyzed, and the lesions were divided into 61 cases in the AFP-negative HCC group and 38 cases in the OM group according to the pathological findings, the CEUS features of the lesions were analyzed and the lesions were classified according to the CEUS LI-RADS v2017. Comparison of CEUS features between the two groups was performed using the χ^2^ test. The sensitivity, specificity, positive predictive value, negative predictive value, and coincidence rate of CEUS LI-RADS v2017 for the diagnosis of AFP-negative HCC and OM were calculated using pathological findings as the gold standard. Results: The differences in features of arterial phase enhancement and wash-out between the HCC and OM groups were statistically significant (*p* < 0.05). The sensitivity of diagnosing HCC by LR-5 was 62.3% and the specificity was 92.1%. The sensitivity of diagnosing OM by LR-M was 92.1% and the specificity was 83.6%. Conclusions: When AFP is negative in patients with intrahepatic focal lesions, LR-5 has high specificity but low sensitivity in the diagnosis of HCC, and LR-M has high sensitivity and specificity in the diagnosis of OM. CEUS LI-RADS is a tool to differentiate AFP-negative HCC and OM effectively.

## 1. Introduction

Primary malignancies of the liver include primary hepatocellular carcinoma (HCC), intrahepatic-cholangiocarcinoma (ICC), combined hepatocellular–cholangiocarcinoma (CHC) and other rare types of tumors. Primary liver cancer has a high incidence and the third highest mortality rate among malignant tumors [1]. HCC accounts for approximately 80–90% of all primary liver cancers, and most patients are found to be in advanced stages with poor prognosis [2,3]. Early HCC can be radically treated by surgery, TACE (transcatheter arterial chemoembolization), and ablation, while the diagnosis and cure of other primary hepatic malignancy (OM) now still depends on pathology and surgical excision [4,5,6]. Alpha-fetoprotein (AFP) is an important serological marker for early screening of HCC, but since about 30–40% of HCC patients are clinically negative for AFP [7], it is easy to misdiagnose AFP-negative patients as other liver tumors, so early and accurate preoperative diagnosis and differentiation of AFP-negative HCC from OM are particularly important for the selection of clinical treatment options and improvement of prognosis [8]. Previous studies have found that the CEUS LI-RADS classification can predict the risk of developing HCC in high-risk patients with hepatocellular carcinoma. The purpose of this study is to explore the differential diagnostic value of CEUS LI-RADS v2017 for AFP-negative HCC and OM, with the aim of making an early and accurate diagnosis of AFP-negative intrahepatic lesions and providing a more valuable imaging basis for the selection of clinical treatment options.

## 2. Materials and Methods

### 2.1. Patients

The clinical, pathological, and imaging data of patients with primary liver malignant tumors diagnosed by percutaneous liver biopsy or surgical pathology from January 2018 to February 2021 were analyzed retrospectively. The inclusion criteria were as follows: (i) CEUS within 2 weeks prior to percutaneous liver biopsy or surgery. (ii) All patients were eligible for the CEUS LI-RADS criteria for the applicable population [9]. (iii) All patients were negative for serum AFP and all were measured within 1 week before and after the CEUS test. The exclusion criteria were as follows: (i) thrombosis in the vein; (ii) the maximum diameter of the tumor lesion <1 cm; (iii) having received other treatment modalities before the CEUS examination; (iv) multiple tumors.

According to the inclusion and exclusion criteria, a total of 99 patients with primary malignant tumors of the liver were eventually included among the 158 patients. There were 74 males and 25 females, aged from 29 to 82 (56.690 ± 10.584) years. A total of 99 AFP -negative liver masses were included in the study, with a maximum diameter of 1.000~19.800 (5.483 ± 3.106) cm. In accordance with the standard of Laboratory Department of our hospital, the normal value of AFP (chemiluminescence method) is 0~7 ng/mL, and AFP < 7 ng/mL is negative. All patients in this study signed an informed consent before contrast-enhanced ultrasound.

### 2.2. Instruments and Methods

CEUS was performed in low mechanical index mode using Philips IU22/EPIQ 7 (Phillips Medical Systems, Bothell, WA, USA) and Siemens ACUSON Sequoia (Siemens Medical Solutions, Ann Arbor, MI, USA) color Doppler ultrasound diagnostic instruments with convex array probes C5-1 and 5C1 (both at 1–5 MHz), respectively, and the contrast agent was Sulphur hexaflfluoride microbubbles (SonoVue, Bracco Imaging, Milan, Italy).

The patients took horizontal position or left supine position, scanned the liver routinely and selected the best section of the lesion. Then the contrast agent SonoVue was added to 5.0 mL saline to make microbubble suspension, and the microbubble suspension was mixed repeatedly for 30 s. According to the instrument and the body weight of the patients, 1.0–2.0 mL microbubble suspension was withdrawn and injected into the elbow vein followed by tail injection of 5 mL saline. While recording the time, the lesion was dynamically observed in real time for 4–6 min continuously and the images were stored in CEUS mode.

### 2.3. Data Analysis

The images were analyzed by two observers with more than 5 years of experience in the diagnosis of abdominal diseases with CEUS using an independent blinded method, with the observer being informed only of the patient’s high risk of HCC and unknown pathological and clinical laboratory findings, assessing the lesion for the presence of the following signs and classifying the lesion with reference to CEUS LI-RADS v2017 (https://www.acr.org) [9], with a third observer with more than 10 years of experience in the diagnosis of abdominal diseases with CEUS making the final classification when the diagnoses differed between the two.

Features of CEUS include the following: (1) Features of HCC, including not rim and not peripheral discontinuous globular arterial phase hyper-enhancement (APHE), late (>60 s) wash-out, and mild wash-out; and (2) features of LR-M (probably or definitely malignant but not HCC specific), including rim APHE, early (<60 s) wash-out, and marked wash-out.

### 2.4. Statistical Analysis

SPSS Statistics 25.0 (IBM Corporation, Armonk, NY, USA) was applied for statistical analysis. The quantitative data were expressed as $\bar{x}$ ± s and compared using the independent samples *t*-test; the categorical variables were expressed as N (%) and compared using the χ^2^ test. The sensitivity, specificity, positive predictive value, negative predictive value, and diagnostic compliance rate were calculated for each CEUS feature and LI-RADS v2017 (https://www.acr.org) for the diagnosis of AFP-negative HCC or OM, using pathological findings as the gold standard.

## 3. Results

### 3.1. General Information of the Patient and CEUS Features of the Lesions

The 99 AFP—negative intrahepatic primary malignant tumors were divided into HCC group (61 cases) and OM group (38 cases, including 31 cases of intrahepatic cholangiocarcinoma, 2 cases of combined hepatocellular carcinoma and cholangiocarcinoma, 3 cases of lymphoma, 1 case of angiosarcoma and 1 case of spindle cell sarcoma) according to pathological findings. All patients in the HCC group had a history of hepatitis B infection, 32 patients in the OM group had a history of hepatitis B infection, 3 patients had hepatitis C cirrhosis, and the remaining 3 patients had alcoholic cirrhosis. There was a statistically significant difference in gender between the two groups (*p* < 0.05), while there was no statistically significant difference in age (*p* > 0.05).

The maximum diameter of the lesions in the HCC group ranged from 1.000 to 19.800 (averaged 5.003 ± 3.337) cm, while the maximum diameter of the lesions in the OM group ranged from 1.700 to 11.900 (averaged 6.253 ± 2.552) cm. After grouping the lesions at a maximum diameter of 3 cm, there was a statistical difference between the two groups in terms of maximum diameter (*p* < 0.05), while there was no statistical difference in terms of location of the lesions (*p* > 0.05). The CEUS features of the lesions in both groups: performance of enhancement in arterial phase and wash-out were statistically different (*p* < 0.05), as shown in Table 1.

### 3.2. Final Results of CEUS LI-RADS Category of Lesions

**Figure 1:**
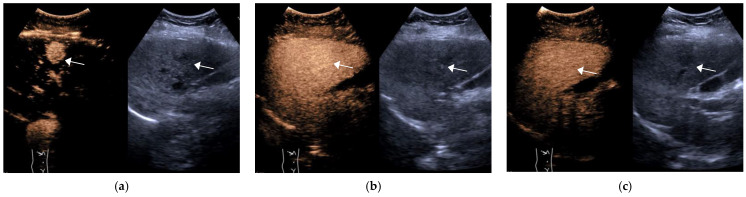
Female, 55 years old, LR-4 category. A slightly hypoechoic lesion of approximately 2.6 × 2.5 cm in size (arrow) is seen in S6, with hyperenhancement in the arterial phase (**a**) and no significant washout in the portal phase (**b**) and delayed phase (**c**). The final pathological diagnosis is highly differentiated HCC.

**Figure 2:**
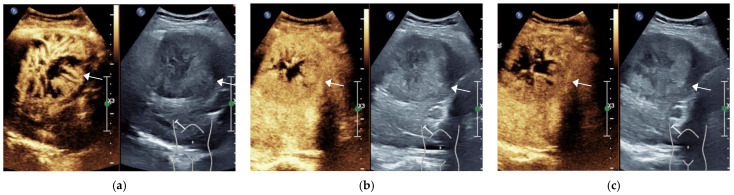
Male, 74 years old, LR-5 Category. A hypoechoic lesion (arrow), approximately 7.7 × 6.7 cm in size, is seen in S5 of the liver, showing inhomogeneous hyperenhancement at 14 s in the arterial phase (**a**), starting to clear at 93 s in the portal phase (**b**) and significantly clearing at 157 s in the delayed phase (**c**). The final pathological diagnosis was moderately differentiated HCC (The white line on the right represents the focus point of the image.).

**Figure 3:**
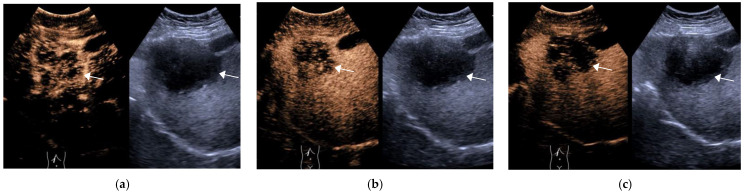
Female, 73 years old, LR-M Category A hypoechoic lesion (arrow), approximately 6.8 × 6.6 cm in size, is seen in S5 of the liver, with peripheral irregular rim enhancement at 15 s in the arterial phase (**a**), starting to wash out at 34 s in the portal phase (**b**) and significantly wash out at 125 s in the delayed phase (**c**). The final pathological diagnosis was hypofractionated ICC.

**Figure 4:**
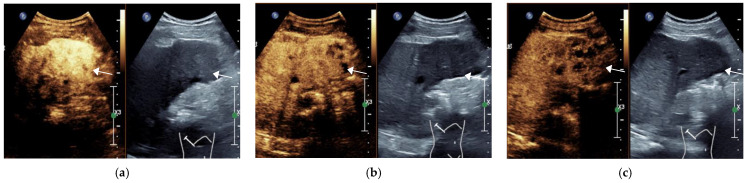
Male, 54 years old, LR-M Category. A hypoechoic lesion (arrow), approximately 5.2 × 5.1 cm in size, is seen in LR-M liver S5, with inhomogeneous hyperenhancement at 23 s in the arterial phase (**a**), starting to wash out at 65 s in the portal phase (**b**) and significantly wash out at 144 s in the delayed phase (**c**). The final pathological diagnosis was combined hepatocellular carcinoma and cholangiocarcinoma. (The white line on the right represents the focus point of the image.)

The results of the CEUS LI-RADS category in the AFP-negative HCC group were 13 cases of LR-4 category, 38 cases of LR-5 category, and 10 cases of LR-M category, and 0, 3, and 35 cases of LR-4 category, LR-5 category, and LR-M category in the OM group respectively (Figure 1, Figure 2, Figure 3 and Figure 4), and there were no patients with LR-1, LR-2, or LR-3 in both groups. The sensitivity, specificity, positive predictive value, negative predictive value, and diagnostic compliance rate of each CEUS feature and CEUS LI-RADS category for the diagnosis of HCC or OM, using pathological findings as the gold standard are as shown in Table 2.

## 4. Discussion

Alpha-fetoprotein (AFP) is an important serological marker for the early screening of hepatocellular carcinoma (HCC), but Berretta et al. [10] found that the specificity of AFP in diagnosing HCC was 76–94%, and the sensitivity was only 39–65%. Therefore, for some AFP negative patients, early diagnosis of the nature of intrahepatic nodules is of great significance for their treatment and prognosis. Contrast-enhanced ultrasound (CEUS) is a convenient, cost-effective, safe and radiation-free imaging tool that can show microvascular perfusion of tumors and is of great value in the early diagnosis and differential diagnosis of HCC. However, there is an overlap between the typical enhancement patterns of HCC and ICC, and there is a risk of misdiagnosis, especially in cirrhosis [11]. Therefore, the guidelines published by the American Association for the Study of Liver Diseases (AASLD) in 2011 and the World Federation of Ultrasound in Medicine and Biology (WFUMB) and European Federation of Societies for Ultrasound in Medicine and Biology (EFSUMB) in 2012 do not consider CEUS as a recommended imaging method for the diagnosis of focal liver lesions in the background of cirrhosis [12]. However, with the development of CEUS, more and more studies [13] have demonstrated that CEUS showing a high diagnostic accuracy in assessing HCC and its diagnostic efficacy is comparable to that of enhanced CT/MRI. The Contrast-enhanced Ultrasound Liver Imaging Reporting and Data System [9] (CEUS LI-RADS) was produced by the American college of radiology (ACR) in 2016 to standardize the image acquisition, reporting, and data collection of CUES examinations for focal intrahepatic lesions in people at high risk of HCC, and it has been updated in 2017. This classification system classifies a category of malignant lesions that are clearly or probably non-HCC as LR-M category. This category may be intrahepatic cholangiocarcinoma (ICC), combined hepatocellular carcinoma and cholangiocarcinoma (CHC), metastases or other primary malignancies of the liver.

The results of this study found that the LR-5 category had a high diagnostic specificity of 92.1% for AFP-negative HCC, but a low sensitivity of 62.3%. Compared to the results of previous studies that did not limit the level of AFP in liver lesions [14,15], the diagnostic efficacy of the CEUS LR-5 category for HCC was reduced when the AFP of intrahepatic focal lesions were negative. Some lesions were classified as LR-4 category due to the lack of significant wash-out in both the portal and delayed phases, resulting in fewer cases being enrolled in LR-5 category, which may be related to the degree of differentiation of the lesions [16]. Feng Y et al. showed [17] that the enhancement pattern and wash-out behavior of HCC correlated with the degree of cellular differentiation of the lesions, with 73.7% of the HCC lesions without wash-out being highly differentiated and 26.3% were moderately differentiated lesions.

The main purpose of the LR-M category is to differentiate ICC and other malignant lesions from HCC. The findings of this study showed that the LR-M category had a high sensitivity and specificity for the diagnosis of OM, 92.1% and 83.6%, respectively. Some studies have shown [14] that the CEUS LI-RADS can distinguish the majority of other liver malignancies from HCC, significantly reducing the possibility of misdiagnosis of ICC as HCC, but also classifying some HCC into the LR-M category. In this study, 10 AFP-negative HCC were classified as LR-M category. It has been proposed [18] that well-differentiated HCC consists mainly of trabecular cell-like cell lines and an abundant blood sinusoidal network, which may lead to ultrasound microbubbles stagnation and slow clearance, resulting in slower or no wash out. In contrast, lower-differentiated HCC grows faster, with distorted neovascularization and accelerated regression of the portal system, resulting in an earlier and more significant wash-out. Therefore, it caused some HCC to be classified as LR-M category, while marked wash-out had a high diagnostic specificity for OM, up to 95.1%. For CHC, the arterial phase enhancement pattern depends on the proportion of HCC and ICC components within the tumor. Some authors [19] summarized the CEUS pattern of CHC, suggesting that most of the hepatocellular carcinoma-dominant type showed diffuse inhomogeneous enhancement and most of the cholangiocellular carcinoma-dominant type showed peripheral irregular rim enhancement. The two cases of CHC in this study both showed non-rim enhancement and late wash-out and were eventually classified as LR-5 rather than LR-M category. Therefore, CEUS LI-RADS has limited diagnostic value for CHC and needs to be combined with other indicators for further diagnosis.

There was also a significant difference in CEUS characteristics between OM and HCC patients (*p* < 0.05). A total of 45 cases of LR-M category lesions were enrolled in this study, of which 10 were AFP-negative HCCs, only one showed rim enhancement in arterial-phase and the rest were non-rim enhancement, while 22 of the remaining 35 cases of OMs showed rim enhancement in the arterial phase. The specificity of arterial-phase rim enhancement for the diagnosis of OM was up to 96.7%, but the sensitivity was low at 57.9%. In comparison, the arterial-phase rim enhancement has high diagnostic sensitivity for AFP-negative HCC, up to 95.1%, but low specificity, only 63.2%, that is, about 40% of primary other types of malignancies of the liver are misclassified as HCC, mainly because other hypervascular lesions of the liver often show this feature, such as hepatic angiomyolipoma [20]. Zou et al. [21] have found that epithelioid angiomyolipoma (EAML) is more easily misdiagnosed as AFP-negative HCC because hepatic EAML shows enhancement patterns that are similar to those of AFP -negative HCC. Therefore, the diagnostic efficacy of non-rim enhancement in the arterial phase for HCC needs to be further explored in the future.

This study has the following limitations:(i) The correlation between the degree of differentiation and CEUS LI-RADS was not analyzed in this study; (ii) there were no LR-1 category, LR-2 category, or LR-3 category lesions in this study, and the results may be somewhat biased; (iii) the diagnostic performance of the ancillary features of CEUS LI-RADS was not analyzed in this study and could be further studied in the future.

## 5. Conclusions

In summary, CEUS LI-RADS have high application value for the differentiation of HCC and OM when AFP-negative patients present with high risk of HCC. The LR-5 category has high specificity but low sensitivity for the diagnosis of HCC, while the LR-M category has high sensitivity, specificity, and diagnostic compliance for the diagnosis of OM. CEUS LI-RADS can be used to identify focal intrahepatic lesions at an early stage, providing clinicians with a new imaging basis for individualized treatment. However, CEUS LI-RADS v2017 still needs to be constantly refined to improve its diagnostic and differential diagnostic efficiency.

## Figures and Tables

**Table 1:** General patient information and CEUS features of the lesions.

**Group**	**Num.**	**Gender**		**Age/Years**		**Location**		**Maximum Diameter/cm**		
		**Male**	**Female**			**Left Lobe**	**Right Lobe**	**<3**		**≥3**
HCC	61	50 (82.0)	11 (18.0%)	55.540 ± 10.096		15 (24.6%)	46 (75.4%)	23 (37.7%)		38 (62.3%)
OM	38	24 (63.2%)	14 (36.8%)	58.530 ± 11.215		11 (28.9%)	27 (71.1%)	3 (7.9%)		35 (92.1%)
X^2^/t	-	4.389		−1.338		0.230		10.744		
*p*	-	0.036		0.185		0.632		0.001		
**Group**	**Num.**	**Enhancement Pattern in AP**			**Degree of Wash-Out**			**Time to Wash Out/s**		
		**Non-Rim APHE**	**Rim** **APHE**	**Non APHE**	**Mild**	**Marked**	**Non**	**<60**	**≥60**	**Non-Wash Out**
HCC	61	58 (95.1%)	2 (3.3%)	1 (1.6%)	54 (88.5%)	3 (4.9%)	4 (6.6%)	11 (18.0%)	46 (75.4%)	4 (6.3%)
OM	38	14 (36.8%)	22 (57.9%)	2 (5.3%)	13 (34.2%)	25 (65.8%)	0 (0%)	32 (84.2%)	6 (15.8%)	0 (0%)
X^2/^t	-	40.745			43.373			41.946		
*p*	-	<0.001			<0.001			<0.001		

HCC = hepatocellular carcinoma, OM = other primary malignancies, APHE = arterial phase hyperenhancement, AP = arterial phase. - = No comparison of the number of cases between the two groups.

**Table 2:** Diagnostic efficacy of CEUS features and CEUS LI-RADS for HCC and OM [% (N)].

	Group	Sen	Spe	PPV	NPV	DCR
non- rim APHE	HCC	95.1 (58/61)	63.2 (24/38)	80.6 (58/72)	88.9 (24/27)	82.8 (82/99)
rim APHE	OM	57.9 (22/38)	96.7 (59/61)	91.7 (22/24)	78.7 (59/75)	81.8 (81/99)
mild wash-out	HCC	88.5 (54/61)	65.8 (25/28)	80.6 (54/67)	78.1 (25/32)	79.8 (79/99)
marked wash-out	OM	65.8 (25/38)	95.1 (58/61)	89.3 (25/28)	81.7 (58/71)	83.8 (83/99)
late wash-out	HCC	75.4 (46/61)	84.2 (32/38)	88.5 (46/52)	68.1 (32/47)	78.8 (78/99)
early wash-out	OM	84.2 (32/38)	82.0 (50/61)	74.4 (32/43)	89.3 (50/56)	82.8 (82/99)
LR-4	HCC	21.3 (13/61)	97.4 (38/39)	100.0 (13/13)	44.2 (38/86)	51.5 (51/99)
LR-5	HCC	62.3 (38/61)	92.1 (35/38)	92.7 (38/41)	60.3 (35/58)	73.7 (73/99)
LR-M	OM	92.1 (35/38)	83.6 (51/61)	77.8 (35/45)	94.4 (51/54)	86.9 (86/99)

HCC = hepatocellular carcinoma, OM = other primary malignancies, APHE = arterial phase hyper-enhancement. Sen = Sensitivity, Spe = Specificity, PPV = Positive predictive value, NPV = Negative predictive value, DCR = diagnostic compliance rate.

## Data Availability

The data that support the findings of this study are available from the corresponding author upon reasonable request.
